# Effect of Novel Marine Nutraceuticals on IL-1**α**-Mediated TNF-**α** Release from UVB-Irradiated Human Melanocyte-Derived Cells

**DOI:** 10.1155/2011/728645

**Published:** 2011-09-22

**Authors:** Visalini Muthusamy, Lynn D. Hodges, Theodore A. Macrides, Glen M. Boyle, Terrence J. Piva

**Affiliations:** ^1^School of Medical Sciences, RMIT University, P.O. Box 71, Bundoora, VIC 3083, Australia; ^2^Drug Discovery Group, Division of Cancer and Cell Biology, Queensland Institute of Medical Research, Herston, QLD 4006, Australia

## Abstract

UV-induced inflammation and reactive oxygen species formation are involved in the development of melanoma. Natural products like 5**β**-scymnol and CO_2_-supercritical fluid extract (CO_2_-SFE) of mussel oil contain anti-inflammatory and antioxidant properties that may aid in reducing the deleterious effects of UV radiation. Therefore, their effect on the release of the proinflammatory cytokine, tumour necrosis factor-**α** (TNF-**α**), from UVB-irradiated human melanocytic cells was examined. Human epidermal melanocytes (HEM) and MM96L melanoma cells were exposed to UVB radiation and IL-1**α**. Cell viability and TNF-**α** levels were determined 24 hours after-irradiation while p38 mitogen-activated protein kinase (MAPK) activation was observed at 15 min after-irradiation. When **α**-tocopherol, CO_2_-SFE mussel oil, and 5*β*-scymnol were added to the UVB-irradiated HEM cells treated with IL-1**α**, TNF-**α** levels fell by 53%, 65%, and 76%, respectively, while no inhibition was evident in MM96L cells. This effect was not due to inhibition of the intracellular p38 MAPK signalling pathway. These compounds may be useful in preventing inflammation-induced damage to normal melanocytes.

## 1. Introduction

The sun emits different types of ultraviolet (UV) light. Our skin is a natural target of UV radiation which is involved in vitamin D_3_ production in our body. UV radiation at high doses is an environmental carcinogen which can elicit skin damage as well as induce skin cancer [1]. It can mediate inflammatory and immunological reactions through activation of receptors, DNA/RNA damage, and production of reactive oxygen species (ROS) [2, 3]. It is also involved in the release of pro-inflammatory cytokines, of which tumour necrosis factor *α* (TNF-*α*) has been implicated in tumorigenic activities [4]. In order to mediate its effects, UV radiation is known to activate multiple signalling cascades such as the p38 mitogen-activated protein kinase (MAPK), c-Jun terminal kinase (JNK), extracellular signal-regulated kinase 1/2 (ERK1/2), and nuclear factor-*κ*B (NF*κ*B) pathways in skin cells [1, 5–7]. The role each of these pathways plays in mediating the release of cytokines such as TNF-*α* remains to be fully characterised. 

The risks of photoageing and skin carcinogenesis may be lowered through the modulation of epidermal inflammation caused by UV-activated cell signalling pathway and/or generation of oxidative stress. It is likely that exogenous anti-inflammatory/antioxidants agents may alter pathway activities. The application of natural products have shown efficacy in reducing inflammation and oxidative stress. Sharma et al. [8] showed that dietary grape seed proanthocyanidins (GSP) markedly decreased UVB-induced (1.2 kJ/m^2^) activation of the NF*κ*B pathway, inducible nitric oxide synthase (iNOS), cycloxygenase-2 (COX-2), and cyclin D1 protein expression in SKH-1 hairless mice. GSP was also found to prevent the depletion of intracellular antioxidants, inhibit UVB-induced ROS production, and activation of MAPK proteins. A root extract of *Lithospermum erythrorhizon* was found to inhibit UVB-induced (0.27 kJ/m^2^) apoptosis and suppressed cytokine production (TNF-*α*, IL1, IL6, and IL8) via the p53 pathway in primary human keratinocytes [9]. Anthocyanins extracted from black soybeans inhibited the NF*κ*B pathway activation and reduced COX and prostaglandin E_2_ (PGE_2_) production in UVB-irradiated hairless mice [10]. These pathways are also involved in proinflammatory cytokine release like TNF-*α* which was shown to be involved in melanoma progression via the inhibition of apoptosis [4, 11]. Therefore, the use of natural products to reduce inflammation may be beneficial in decreasing the deleterious effects of these cytokines. 

Topical application of *α*-tocopherol and *α*-tocopheryl succinate reduced skin inflammation, pigmentation, and incidence of skin cancer in SKH-2 hairless mice [12]. *α*-Tocopherol has been shown to reduce both cyclobutane pyrimidine photoproducts on UV exposed skin [13] and the incidence of UV-induced tumours in mice [14]. Besides its anti-inflammatory property, it has been shown to act as an antioxidant by reducing UV/ROS-induced damage in human and mouse skin cells [8, 15–17]. Its antioxidant property is derived from the hydroxyl group present on the aromatic ring which donates its hydrogen to a free radical (e.g., hydroxyl and lipid peroxide radical) to form a nonradical stable product (e.g., water and lipid molecules) [18]. 

Relatively new compounds like CO_2_-supercritical fluid extraction (SFE) of *Perna canaliculus* mussel oil [19, 20] and 5*β*-scymnol (5*β*-cholestane-3*α*,7*α*,12*α*,24,26,27-hexol) [21, 22] are still being investigated to identify their biological activities which may be beneficial in reducing the risk of UV exposure on skin cells. CO_2_-SFE mussel oil of *P. canaliculus *(Bivalvia: Mytilidae; New Zealand green-lipped mussel from Hallam Cove, New Zealand) has demonstrated anti-inflammatory and antioxidant properties [19, 23]. The mussel oil derived from tartaric acid-stabilized freeze-dried *P. canaliculus* by the CO_2_-SFE method [24] is a unique blend of *n*-3 polyunsaturated fatty acid [25] that includes 7,11,14,17-eicosatetraenoic acid [19], a structural isomer of the pro-inflammatory arachidonic acid. It has been shown to reduce inflammation in a rat adjuvant-induced arthritis model [20, 26]. The antiarthritic effect is explained in part on the basis of modulation of arachidonic acid metabolism (i.e., COX and lipoxygenase (LOX) inhibition). 

5*β*-Scymnol is present in the bile of sharks and stingrays as scymnol-26-sulfate [21]. It is an active component of “deep sea liver oil,” which is used as a Japanese folk remedy to treat skin inflammation resulting from scalds, burns, and acne [27]. 5*β*-Scymnol is a metal ion chelator *in vitro* and scavenger of the hydroxyl radical [21, 28]. Macrides et al. [21] found that 5*β*-scymnol was a more potent hydroxyl scavenger than Trolox (*α*-tocopherol analogue) and marketed pycnogenols preparations extracted from pine tree bark and grape seeds. 5*β*-Scymnol's antioxidant and metal ion chelating properties resides in its tri-alcohol-substituted aliphatic side chain moiety [21]. 

Since UV radiation generates inflammation and oxidative stress in melanocytes which may lead to its transformation into melanoma, this study investigated the effect of the CO_2_-SFE mussel oil and 5*β*-scymnol in relation to *α*-tocopherol on the release of the pro-inflammatory cytokine TNF-*α* via the p38 MAPK pathway in UVB-irradiated human melanocyte-derived cells. 

## 2. Results

### 2.1. Comparison of Antioxidant Activity

It has been shown that UV radiation can deplete antioxidant levels and induce the production of ROS in melanocyte-derived cells which can inturn increase inflammation [29–32]. As such, exogenous sources of antioxidants may be necessary to elevate intracellular antioxidant levels and thereby reduce UV-induced inflammation. Therefore, before comparing the effects of CO_2_-SFE mussel oil and 5*β*-scymnol to that of *α*-tocopherol on UVB-irradiated human melanocytic cell lines (HEM and MM96L), their antioxidant effects were first measured using the DPPH and Fenton assays. *α*-Tocopherol was used as a control in this study because it is lipophilic like that of CO_2_-SFE mussel oil and 5*β*-scymnol. It is also a known antioxidant [18, 33, 34]. Structurally, *α*-tocopherol is unlike that of either CO_2_-SFE mussel oil or 5*β*-scymnol.

### 2.2. 2,2-Diphenyl-1-picrylhydrazyl (DPPH) Assay

**Figure 1:**
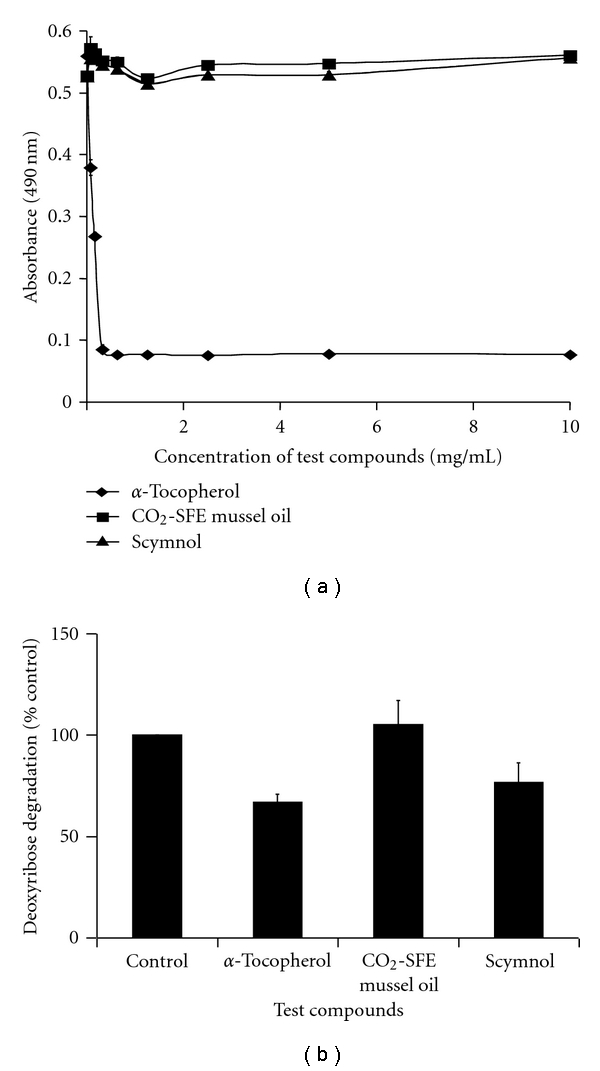
Comparison of the scavenging capacity of the test compounds. (a) DPPH radical scavenging assay. The test compounds (0–10 mg/mL) were added to 100 *μ*M DPPH and absorbance was read at 490 nm after 3 h of incubation with the test compounds. (b) Fenton reaction assay. A comparison of the effect of 10 mg/mL *α*-tocopherol, CO_2_-SFE mussel oil, and scymnol on the degradation of deoxyribose by a Fenton reaction. Absorbance was read at 532 nm. Results expressed as the means ± SD of triplicate samples.

The DPPH assay measures the radical scavenging capacity of antioxidants [35]. Once DPPH accepts an electron or hydrogen radical from an antioxidant, it is reduced to DPPH_2_ which is a clear stable product that loses absorbance at 490 nm. The test compounds used in this study were dissolved in ethanol and as such this carrier solvent was used as a control in this assay. Ethanol did not react with the DPPH assay (results not shown). *α*-Tocopherol (≤10 mg/mL) rapidly quenched the DPPH radical while neither CO_2_-SFE mussel oil nor 5*β*-scymnol (≤10 mg/mL) elicited any effects from 0–30 min (results not shown) and there was no change over a 3 h period (Figure 1(a)).

### 2.3. Fenton Reaction Assay

In the Fenton reaction assay, antioxidants compete with deoxyribose for hydroxyl radicals [36]. The antioxidants were dissolved in ethanol (10 mg/mL) before being assayed. As ethanol reacts with the assay, it was evaporated by the introduction of air. The effect of ethanol (which was evaporated) on deoxyribose degradation was set at 100% (Figure 1(b)). *α*-Tocopherol was shown to be the most protective as it prevented the degradation of deoxyribose by 33% while that of 5*β*-scymnol was 23% protective and CO_2_-SFE mussel oil had no effect (Figure 1(b)). 

### 2.4. The Effect of Test Compounds on the Viability of Melanocyte-Derived Cells

**Figure 2:**
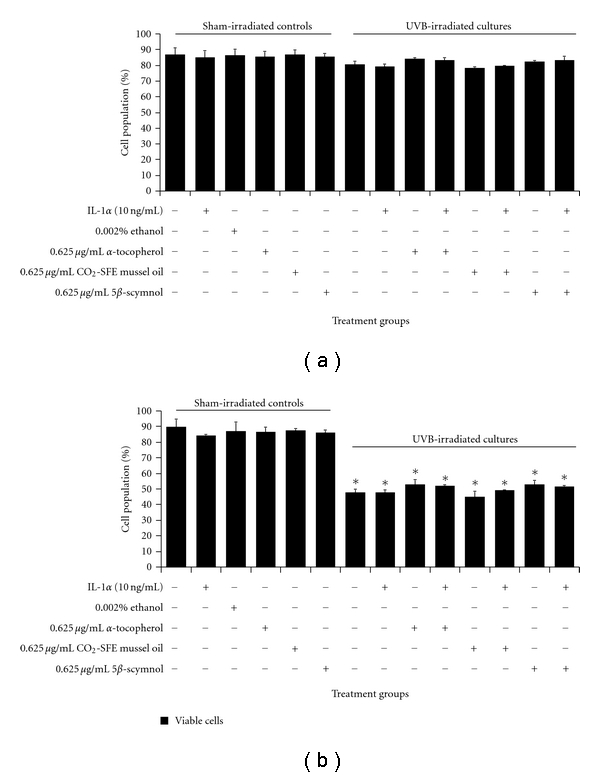
The effect of *α*-tocopherol, CO_2_-SFE mussel oil, and 5*β*-scymnol on the viability of (a) HEM and (b) MM96L cell cultures at 24 h after-UV irradiation. The cells were incubated with the relevant test compounds for 24 h prior to UVB exposure (2 kJ/m^2^). After UVB exposure, the cells were incubated for 24 h with the appropriate test compounds before cell viability was determined using trypan blue solution. Results expressed as the means ± SD of triplicate samples. Comparisons were made between sham-irradiated control and UV-irradiated cultures using Student's paired *t*-test where significance was recorded as *P* ≤ 0.05 (∗).

In the viability study, the test compounds were reconstituted in ethanol. This solvent had no effect on sham- and UVB-irradiated HEM cell viability (Figure 2(a)). Initially, three different concentrations (0.625, 6.25, and 62.5 *μ*g/mL) of test compounds were examined and the two higher doses (6.25 and 62.5 *μ*g/mL) of CO_2_-SFE mussel oil, and 5*β*-scymnol were found to be highly toxic as all the MM96L cells died in culture [1]. In the sham-irradiated HEM cultures, 0.625 *μ*g/mL of *α*-tocopherol, CO_2_-SFE mussel oil, and 5*β*-scymnol had no effect on the number of attached viable cells (~86%; Figure 2(a)). A similar observation was seen in the UVB-irradiated HEM cells where neither *α*-tocopherol, CO_2_-SFE mussel oil, nor 5*β*-scymnol had an effect on the number of attached viable cells even if interleukin-1*α* (IL-1*α*) was present (Figure 2(a)). 

In the MM96L cultures, ethanol had no effect on the viability of either sham- or UVB-irradiated controls (Figure 2(b)). None of the test compounds (*α*-tocopherol, CO_2_-SFE mussel oil, and 5*β*-scymnol) affected the viability of the adhered cell fraction when they were added to the sham-irradiated cells. The viability of UVB-irradiated MM96L cells was significantly less than that of the sham-irradiated controls (Figure 2(b)). The addition of the test compounds to the irradiated cells had no effect on cell viability (Figure 2(b)). The addition of IL-1*α* also had no effect on the cell viability of the treated irradiated cells. Therefore, it can be seen that at the doses used, these test compounds had no significant effect on the viability of either HEM or MM96L cells under the conditions tested.

### 2.5. The Effect of Test Compounds on UV-Induced TNF-*α* Release in Melanocyte-Derived Cells

**Figure 3:**
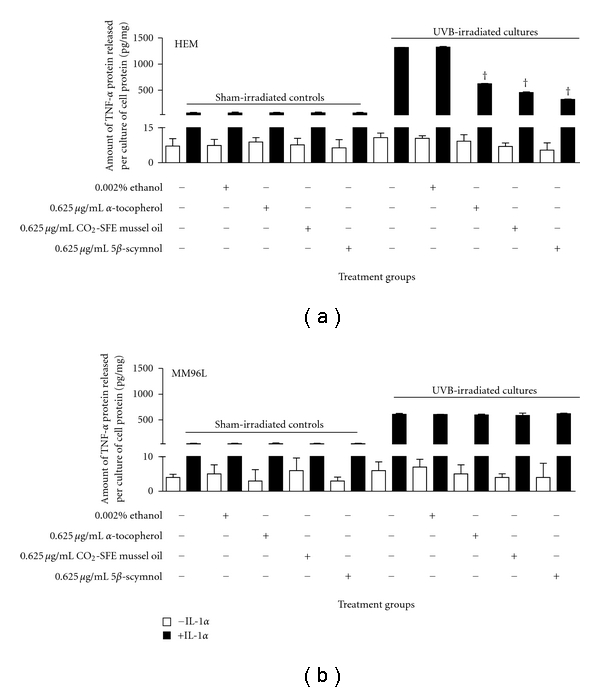
Effect of *α*-tocopherol, CO_2_-SFE mussel oil, and 5*β*-scymnol on TNF-*α* release in UVB-irradiated (a) HEM and (b) MM96L cells. Cell cultures were incubated with 0.625 *μ*g/mL of test compounds for 24 h prior to and after UVB-irradiation (2 kJ/m^2^) with or without 10 ng/mL IL-1*α*. After incubation, the media samples were collected and microconcentrated for ELISA, and the cell lysates were used to determine total protein concentration. Results were expressed as TNF-*α* released (pg)/cell protein (mg) and represent means ± SD of triplicate samples. Statistical analysis was performed using Student's paired *t*-test where significance was recorded as *P* ≤ 0.05. Significant difference between UVB-irradiated untreated sample and test compound-treated irradiated sample in the (∗) absence of IL-1*α* or (†) presence of IL-1*α*.

TNF-*α* may be involved in anti- or protumour activities in melanoma development [11, 37]. Ivanov and Ronai [11] found that TNF-*α* promoted cell survival of LU125 melanoma cells as ATF 2-mediated suppression of TNF-*α* expression led to UVC-induced (0.06 kJ/m^2^) susceptibility to apoptosis. Therefore, the efficacy of these compounds in inhibiting TNF-*α* release in melanocyte-derived cells was investigated. In the sham-irradiated HEM cells, the level of TNF-*α* released was low (7 pg/mg cell protein) and the addition of the test compounds had no effect on these levels (Figure 3(a)). UVB radiation did not induce a significant increase in TNF-*α* release from HEM cells (11 pg/mg cell protein; Figure 3(a)). Following the addition of test compounds, the TNF-*α* levels were lower than that seen for the untreated irradiated cells. When IL-1*α* (10 ng/mL) was added to the UVB-irradiated melanocyte cultures, there was an increase in TNF-*α* release (120-fold; Figure 3(a), Table 1) similar to that seen in cultured keratinocytes (results not shown) and previous studies [38–40]. When the IL-1*α* stimulated melanocyte cells were treated with *α*-tocopherol, CO_2_-SFE mussel oil, and 5*β*-scymnol, the levels of TNF-*α* shed from the cells fell by 53%, 65%, and 76%, respectively (Figure 3(a), Table 1).

In MM96L cells, the addition of the test compounds had no effect on the levels of TNF-*α* released from the unirradiated cells (Figure 3(b)). When IL-1*α* was added to the UVB-irradiated cells, a 101-fold increase in the level of TNF-*α* released from the cells was observed (Figure 3(b), Table 1). Of interest was that the test compounds had no effect on the level of TNF-*α* released from the IL-1*α* stimulated UVB-irradiated cells (Figure 3(b), Table 1). 

### 2.6. The Effect of Test Compounds on the Activation of p38 MAPK Pathway in UV-Irradiated Melanocyte-Derived Cells

**Figure 4:**
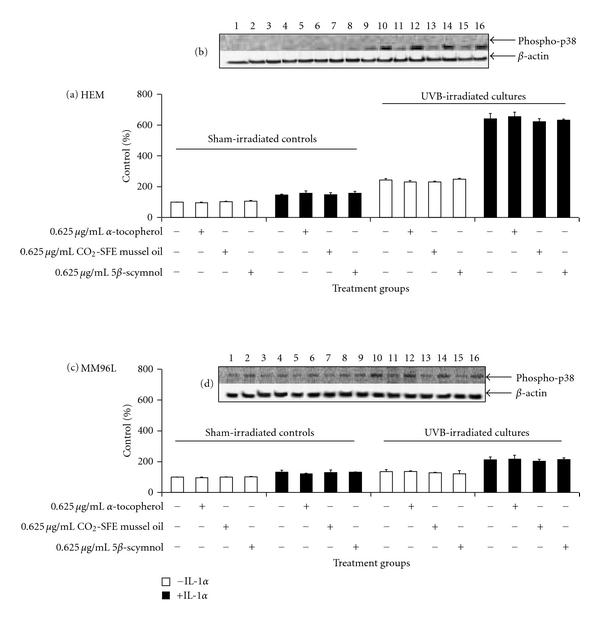
Effect of test compounds on the expression of phospho-p38 protein in UVB-irradiated (2 kJ/m^2^) (a and b) HEM and (c and d) MM96L cells. Cell cultures were incubated with 0.625 *μ*g/mL of test compounds for 24 h prior to and 15 min after UVB-irradiation with or without 10 ng/mL IL-1*α*. The proteins were extracted at 15 min after-irradiation and western blotting was performed. A representative western blot probed for phospho-p38 MAPK in (b) HEM and (d) MM96L cells incubated with either *α*-tocopherol, CO_2_-SFE mussel oil, or 5*β*-scymnol prior to and after UVB-irradiation. The sham-irradiated control (lanes 1–8) or cell cultures exposed to high-dose UVB radiation (lanes 9–16) were treated with 10 ng/mL IL-1*α* (lanes 2, 4, 6, 8, 10, 12, 14, and 16). Lanes 1, 2, 9, 10: no test compound, lanes 3, 4, 11, 12: 0.625 *μ*g/mL *α*-tocopherol, lanes 5, 6, 13, 14 : 0.625 *μ*g/mL CO_2_-SFE mussel oil, and lanes 7, 8, 15, 16: 0.625 *μ*g/mL 5*β*-scymnol. Results expressed as the means ± SD of triplicate samples. Statistical analysis was performed using Student's paired *t*-test where significance was recorded as *P* ≤ 0.05. (∗) Significant difference between untreated sample and test compound-treated sample.

The p38 inhibitor, SB202190, inhibited TNF-*α* release in UVB-irradiated HEM and MM96L cells [1]. As *α*-tocopherol, CO_2_-SFE mussel oil and 5*β*-scymnol suppressed UV-induced TNF-*α* release, it was of interest to observe if these compounds also inhibited the activities of p38 MAPK. In both sham-irradiated HEM and MM96L cells, endogenous phospho-p38 expression was shown to be minimal (Figure 4). However, within 15 min exposure to UVB-irradiation, phospho-p38 protein expression was increased in both cell lines [1]. When IL-1*α* was added to the irradiated cells, the increase in phospho-p38 levels was higher in HEM (640% versus 242% in untreated UVB-irradiated HEM cells) than in MM96L (213% versus 134% in untreated UVB-irradiated MM96L cells) cells. The test compounds had no effect on phospho-p38 levels in either the sham- or UV-irradiated HEM and MM96L cells. This suggests that these compounds do not inhibit TNF-*α* release in HEM cells by suppressing p38 MAPK activity.

## 3. Discussion

It can be seen from the DPPH and Fenton reaction assays that *α*-tocopherol was able to reduce DPPH and scavenge hydroxyl radicals whereas 5*β*-scymnol was only able to scavenge hydroxyl radicals (Figure 1). Although 5*β*-scymnol scavenged hydroxyl radicals, it did not inhibit ferrous-induced lipid peroxidation in rat hepatocytes [28]. CO_2_-SFE mussel oil on the other hand did not react in any of these assays which suggests that it is unable to scavenge either radicals (Figure 1). Whitehouse et al. [26] and Halpern [41] reported that CO_2_-SFE mussel oil contains carotenoids which are antioxidants known to scavenge singlet oxygen and peroxyl radicals. CO_2_-SFE mussel oil was shown to inhibit LOX metabolites which suggest that it can reduce LOX-mediated lipid peroxidation [42]. However, the ability of these compounds to scavenge oxidants did not correlate with their effect on HEM and MM96L cell viability. 

HEM cells were less susceptible to UVB than were MM96L cells. The percentage of attached viable cells following UV radiation was higher in HEM cells than in MM96L cells (Figure 2). This suggests that HEM cells may either have a more efficient DNA repair mechanism or a higher melanin content than MM96L cells. Furthermore, it was found that melanoma cells were more susceptible to UVB radiation via XIAP degradation and caspase 3 upregulation [43]. In this study, *α*-tocopherol, CO_2_-SFE mussel oil, or 5*β*-scymnol at 0.625 *μ*g/mL had no significant cytotoxic effects on HEM and MM96L cells (Figure 2). However, CO_2_-SFE mussel oil and 5*β*-scymnol were cytotoxic to MM96L cells when doses greater than 0.625 *μ*g/mL were used. At 0.625 *μ*g/mL, these compounds did not confer significant protection from UVB-induced cell death, and suggests that either (a) free radicals may not be involved in this process or (b) these compounds did not affect the cell death pathways. 

*In vitro* studies have showed that keratinocytes, fibroblasts, and skin equivalents can produce IL-1*α* in response to UV radiation [44–46]. IL-1*α* can initiate cell signalling in a paracrine/autocrine fashion on neighbouring cells like melanocytes expressing IL1 receptors [47, 48]. Therefore, the addition of exogenous IL-1*α* may simulate a similar situation in an *in vitro* study in the absence of other IL-1*α* secreting skin cell types. Bashir et al. [38] found that IL-1*α* upregulated TNF-*α* levels in UVB-irradiated keratinocytes. In our study, although UV radiation alone did not induce high levels of TNF-*α* release, the addition of IL-1*α* further enhanced these levels from UVB-irradiated HEM (120-fold) and MM96L (101-fold) cells (Table 1). Interestingly, in irradiated HEM cells, TNF-*α* release was approximately twice that observed in MM96L cells (Figure 3). This may suggest that high levels of inflammation may not be necessary for cells which have acquired malignancy. 

When the test compounds were added to UVB-irradiated HEM cells treated with IL-1*α*, inhibition of TNF-*α* released from these cells was observed (Figure 3). 5*β*-Scymnol (76%) was the most effective in inhibiting UVB-induced TNF-*α* release followed by CO_2_-SFE mussel oil (65%) and *α*-tocopherol (53%). However, in the IL-1*α* stimulated UVB-irradiated MM96L cells, all three compounds were unable to suppress UVB-induced TNF-*α* release. It is possible that different mechanisms may be involved in UV-induced TNF-*α* release in melanoma and melanocyte cells and as such these test compounds may inhibit certain mechanisms but not others resulting in varying efficacy of inhibition. Pupe et al. [49] showed that in keratinocytes, the antioxidants, butylated hydroxyanisole (BHA; 200 *μ*M) completely inhibited UVB-induced (0.3 kJ/m^2^) TNF-*α* release; 3 mM N-acetylcysteine (NAC) induced a 2.5-fold inhibition; epigallocatechin gallate (EGCG; 50 *μ*M) and vitamin C (1 mM) had minimal effect while vitamin E (50 *μ*M) had no effect. BHA is a LOX inhibitor as well as a free radical scavenger and as such BHA could have caused a complete suppression of TNF-*α* due to its free radical quenching capacity as well as inhibition of LOX which may be a mechanism distinct from that inhibited by the other antioxidants used [49]. 

In this study, CO_2_-SFE mussel oil inhibited UVB-induced TNF-*α* release in HEM cells (Figure 3). It has been suggested that CO_2_-SFE mussel oil may possess both antioxidant and anti-inflammatory properties as it contains polyunsaturated fatty acids and carotenoids [26, 41]. Since TNF-*α* is a pro-inflammatory cytokine which is produced by the COX pathway in response to UV, it is likely that CO_2_-SFE mussel oil inhibits this release via its anti-inflammatory property [49]. Sandoval et al. [50] found that in murine macrophages, cat's claw inhibited lipopolysaccharide-induced TNF-*α* release at doses lower than that required for its antioxidant activity. Therefore, it is possible that CO_2_-SFE mussel oil may need to be added at higher concentrations to exhibit antioxidant activity in HEM cells. While *α*-tocopherol and 5*β*-scymnol were also able to inhibit TNF-*α* release, it is not clear if this effect is due to their antioxidant or anti-inflammatory properties. Further studies would need to be undertaken to confirm if this inhibitory effect on TNF-*α* release is mediated at the transcriptional, posttranscriptional, or protein secretion level in HEM cells.

It has been shown that p38 MAPK is upregulated in UVB-irradiated skin cells [1]. Ivanov et al. [37] found that inhibition of p38 MAPK pathway led to a decrease in TNF-*α* transcriptional activation. Since these compounds inhibited TNF-*α* release in HEM cells, their effect on p38 MAPK pathway activity was examined (Figure 4). Activation of the p38 MAPK pathway did not appear to be affected by the addition of any of the test compounds in the UVB-irradiated HEM and MM96L cells. In contrast, previous studies have shown that the p38 MAPK pathway can be activated by free radicals and as such antioxidants targeting these oxidants should also inhibit the activation of this pathway [16, 51]. When UVB (4 kJ/m^2^) or UVC-irradiated (0.06 kJ/m^2^) JB6 C141 mouse epidermal cells were treated with catalase, it was shown that H_2_O_2_ activated both the p38 MAPK and JNK pathways in these cells [51]. Larsson et al. [52] found that *α*-tocopherol prevented the loss of glutathione and the translocation of (NF*κ*B) subunit into the nucleus of UVB- (0.6 kJ/m^2^) and UVA-irradiated (60 kJ/m^2^) melanocytes. Since little is known on the effect of CO_2_-SFE mussel oil or 5*β*-scymnol on signal transduction pathways, their effects on other pathway intermediates such as JNK, NF*κ*B, and ERK in the UV-irradiated cells will be investigated in future studies. 

In general, the test compounds inhibited the release of TNF-*α* from UVB-irradiated HEM cells but not by suppressing p38 MAPK pathway activity. This suggests that these compounds mediate their effects via other signalling pathways. Moreover, while 5*β*-scymnol and CO_2_-SFE mussel oil exhibit potential anti-inflammatory and antioxidant properties, they or other nutraceuticals may be useful supplements to add to sunscreens. These compounds may help reduce the detrimental effects of UV radiation in the skin by protecting these cells from inflammatory and/or oxidative stress as a result of exposure. Further research on the mechanisms inhibited by 5*β*-scymnol and CO_2_-SFE mussel oil in UV-irradiated skin cells is currently being investigated. 

## 4. Materials and Methods

### 4.1. Materials

The following chemical and biochemicals: RPMI medium 1640, medium 254, human melanocyte growth supplement, penicillin-streptomycin-glutamine, PBS (phosphate-buffered saline), trypsin-EDTA solution and phenol-red free HBSS (Hank's buffered salt solution) were obtained from Invitrogen (Melbourne, Australia); FBS (foetal bovine serum) and BSA (bovine serum albumin) were from Bovogen (Melbourne, Australia); Chemilucent kit, Goat HRP conjugated antirabbit immunoglobin, and antimouse immunoglobin were from Millipore (Sydney, Australia); Phospho-p38 rabbit polyclonal antibody was from Genesearch (Gold Coast, Australia); AccuKine Human TNF-*α* ELISA Kit was from Scientifix (Melbourne, Australia). All other chemicals were from Sigma (Sydney, Australia), unless otherwise indicated. All tissue culture vessels were obtained from Diethelm Keller SiberHegner (DKSH, Melbourne, Australia); while Microcon YM-10 micro-concentrators (10 kDa) were from Millipore (Sydney, Australia).

### 4.2. Cell Types

The HEM (human epidermal melanocytes) cells obtained from Banksia Scientific (Brisbane, Australia) and MM96L melanoma cells [53] kindly donated by Dr. Glen Boyle (QIMR, Brisbane, Australia) were grown in culture at 37°C. HEM cells were cultured with medium 254 supplemented with 1% (v/v) human melanocyte growth supplement and 1% (v/v) penicillin-streptomycin-glutamine (10,000 units/mL penicillin G sodium, 10,000 *μ*g/mL streptomycin sulfate and 29.2 mg/mL L-glutamine). The spent culture media was discarded and replaced with fresh media every two to three days. MM96L cells were cultured with RPMI medium 1640 supplemented with 5% (v/v) FBS and 1% (v/v) penicillin-streptomycin-glutamine. Spent culture media was removed and discarded every three to four days and replaced with fresh RPMI media. 

### 4.3. Subculture

When the HEM and MM96L cell cultures reached confluence, the respective spent culture media were aspirated and the cells washed with twice with sterile phosphate-buffered saline (PBS) and once with trypsin-EDTA solution. After which, the cells were incubated with sterile trypsin-EDTA solution and the trypsinized cells were used to seed the petri dishes or 6-well plates used in experiments. All solutions used in tissue culture were kept at 37°C for MM96L cells and at RT (20°C) for HEM cells unless specified otherwise.

### 4.4. UV-Irradiation

The UV cabinet (Wayne Electronics, Sydney, Australia) housed 3 UVB Phillips Ultraviolet 8 TL20 W/01 RS lamps (Phillips, Eindhoven, Holland). The cells were only exposed to UVB radiation (305–315 nm) which had a maximal output at 311-312 nm. The variation in the output (mW/cm^2^) of the UV lamps was measured using a UVB detector (attached to an IL-1400A Photometer (International Light, Newburyport, USA). Kuchel et al. [54] found that the average UV dose to induce 1 MED was 41 ± 2 kJ/m^2^ when exposed to solar-simulated UV light. As it is estimated that the UVB component of sunlight is 5%, we chose a dose of 2 kJ/m^2^ which represents the UVB component of 1 MED of solar sunlight (40 kJ/m^2^). The irradiation protocol was performed as described by Huynh et al. [5]. 

### 4.5. Cell Viability

Cell viability was determined 24 h after-UV radiation using the Trypan Blue exclusion method as previously described [5]. 

### 4.6. Test Compounds

CO_2_-SFE mussel oil was obtained from green-lipped mussels harvested on the south coast of New Zealand. The mussels were stabilized with tartaric acid and freeze dried to yield pulverized dry powder. The concentrated mussel oil was extracted from the powder through supercritical fluid extraction according to the method of Macrides and Kalafatis [24]. The lipid composition of CO_2_-SFE mussel oil was characterised using thin layer and gas chromatography [42]. 5*β*-scymnol was isolated and purified from shark bile salt according to the method of Amiet et al. [22]. ^13^CNMR spectrum of the isolated and purified 5*β*-scymnol was identical to that of Amiet et al. [22]. 

Cells cultured in 60 mm petri dishes were pretreated for 24 h with 0.625 *μ*g/mL of either *α*-tocopherol, CO_2_-SFE mussel oil or 5*β*-scymnol dissolved in ethanol [21, 23, 26]. At the end of this period, the culture media was removed and the cell cultures were washed twice with PBS. After two washes with PBS the cultures were overlayed with Hank's-buffered salt solution (HBSS) and exposed to UVB radiation as described above. Immediately following irradiation, HBSS was removed and the test compounds were readded to the cells and were incubated for various time points as seen in the results section.

### 4.7. DPPH Assay

The DPPH assay was used to determine the ability of the test compounds to scavenging DPPH radicals. The test compounds (*α*-tocopherol, CO_2_-SFE mussel oil, and 5*β*-scymnol) were serially diluted in ethanol to determine at which concentration the test compounds were effective in scavenging the DPPH radical [55]. Once DPPH was added, its absorbance (490 nm) was immediately measured at varying time points over a 24 h period using a Perkin Elmer Victor^3^ plate reader (Wallac, Turku, Finland). 

### 4.8. Fenton Reaction Assay

In order to determine the capacity of these test compound in scavenging hydroxyl radicals, the Fenton reaction assay was performed as described by Macrides et al. [21]. 

### 4.9. Western Blotting

The cells were harvested 15 min after-irradiation. The cells were lysed with ice-cold NETN buffer (100 mM NaCl, 20 mM Tris (pH8), 1 mM EDTA, 0.5% (v/v) BRIJ35, 4% (v/v) protease inhibitor, 1% (v/v) phosphatase inhibitor) [56]. Cell protein lysates were prepared and used for western blotting as described [5]. The nylon membranes were incubated with the relevant antibody (1 : 1000 phospho-p38 rabbit polyclonal antibody, 1 : 1000 *β*-actin (loading control)) overnight at 4°C. After which, they were incubated with the appropriate secondary antibody (1 : 1000 goat HRP conjugated antirabbit immunoglobin). The membranes were developed in Chemilucent solution and visualised using a Chemidox XRS unit (BioRad). The digital image was analysed for densitometry using Quantity One Digital Imaging Software Version 4.5.1 (BioRad). The level of phospho-p38 in control sham-irradiated cells was expressed as 100% and the irradiated cells were expressed as a percentage of this value. 

### 4.10. ELISA

The levels of TNF-*α* released from the UVB-irradiated cell cultures were measured 24 h after-irradiation. Immediately after UV exposure, fresh media were added to the cells. In some experiments, 10 ng/mL of IL-1*α* was added to the media as it stimulates TNF-*α* release from UV-irradiated keratinocytes [38]. Aliquots of the culture media were concentrated using Microcon YM-10 micro-concentrators. The levels of TNF-*α* in these media samples were determined using an AccuKine Human TNF-*α* ELISA Kit. 

### 4.11. Statistical Analysis

The results obtained in this study were expressed as the mean ± standard deviation (SD) from triplicate samples. The statistical significance was determined by the use of Student's paired, one-tailed *t*-test with *P* ≤ 0.05 deemed to be significant.

## Figures and Tables

**Table 1:** Effect of IL-1*α* and test compounds on the release of TNF-*α* from UVB-irradiated HEM and MM96L cell line.

(UVB + IL-1*α*)				
Cell line	Untreated	*α*-Tocopherol	CO_2_-SFE mussel oil	Scymnol
HEM	120*	0.5^+^	0.3^+^	0.2^+^
MM96L	101*	1^+^	1^+^	1^+^

*All values are calculated as the fold increase of UVB + IL-1*α* cells compared to their corresponding UVB-irradiated cells.

^+^All values are calculated as the fold decrease of UVB + IL-1*α* cells treated with test compounds compared to untreated UVB + IL-1*α* cells.
